# Drug Reaction With Eosinophilia and Systemic Symptoms: A Diagnostic Dilemma

**DOI:** 10.7759/cureus.34381

**Published:** 2023-01-30

**Authors:** Nicole M Vecin, Mohamed Elsheshtawi, Mohamed Abdul Qader, Stefanie Furlan, Daniel M Lichtstein

**Affiliations:** 1 Medicine, University of Miami Miller School of Medicine, Miami, USA; 2 Internal Medicine, University of Miami, JFK Medical Center, Atlantis, USA; 3 Internal Medicine, University of Miami Miller School of Medicine, Miami, USA

**Keywords:** atypical presentation, drug reaction with eosinophilia and systemic symptoms (dress), delayed diagnosis, cutaneous adverse drug reaction, drug reaction with eosinophilia and systemic symptoms

## Abstract

Drug reaction with eosinophilia and systemic symptoms (DRESS) is an adverse reaction to medications such as sulfonamide-containing antibiotics, anticonvulsants, vancomycin, and non-steroidal anti-inflammatory drugs (NSAIDs). It typically presents with a characteristic rash, eosinophilia, and visceral organ failure. Patients who do not present with characteristic features of DRESS are at risk for delayed diagnosis and treatment. Early diagnosis of DRESS is imperative in preventing unfavorable outcomes such as multi-organ involvement and death. This case report presents the case of a patient who was diagnosed with DRESS but did not display a classic presentation.

## Introduction

Drug reaction with eosinophilia and systemic symptoms (DRESS) is a rare but potentially fatal syndrome related to the use of sulfonamide-containing antibiotics, anticonvulsants, vancomycin (high-risk drugs), and non-steroidal anti-inflammatory drugs (NSAIDs). Clinical features include a characteristic skin rash, fever, lymphocytosis, eosinophilia, and visceral organ involvement, which typically appear two to eight weeks after initiation of one of the aforementioned drugs [1,2,3]. The most predominant clinical features of DRESS, as determined by a retrospective review, include cutaneous eruption (100%), malaise (83%), fever (78%), lymphadenopathy (73%), and abnormal liver function (67%) [4]. Traditional diagnostic criteria for DRESS involve the triad of fever, rash, and eosinophilia, but these features may not always be present. The literature reports few cases of DRESS without eosinophilia, although one retrospective review reports that only 52% of DRESS cases presented with eosinophilia [5]. In addition, one case report presents a rare presentation in which a patient with DRESS presented with eosinophilia and visceral organ involvement but no cutaneous findings [6].

Due to the heterogeneity in the presentation of DRESS, diagnosis provides a challenge as symptoms may confound clinicians and obscure the diagnosis. Diagnosis is based on clinical suspicion and the exclusion of other diseases [6]. A 2012 study reports that the mean delay in diagnosis of DRESS was 1.7 days, which was thought to be understated compared to other institutions due to the 24-hour availability of dermatology services at the given institution [4]. This study also reported that presenting symptoms may appear as due to an infectious cause, with 50% of patients initially considered as infectious and were managed with antibiotics, which has the potential to further propagate the disease [4]. Prompt diagnosis and subsequent treatment of DRESS are crucial in preventing multiple organ failure and death.

In this article, we report an atypical presentation of DRESS and seek to prompt clinicians to consider DRESS as part of the differential diagnosis when systemic illness occurs in the setting of the use of high-risk drugs.

## Case presentation

**Figure 1:**
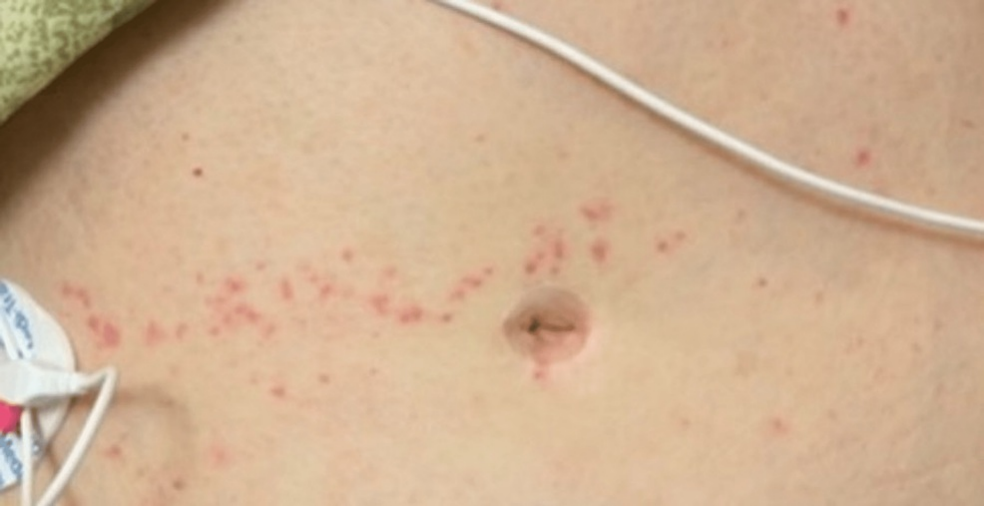
Day one of admission

**Figure 2:**
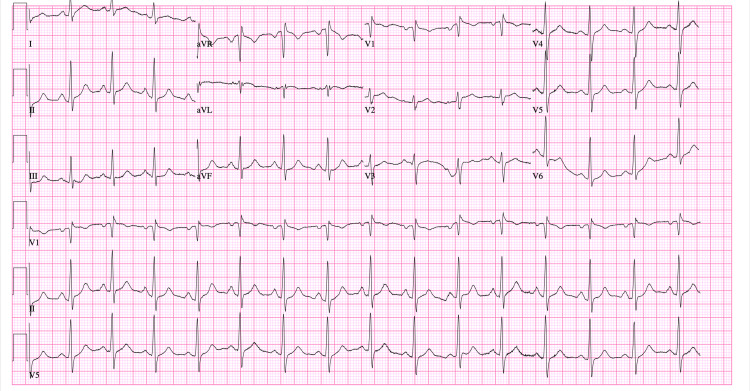
EKG on admission

A 74-year-old woman presents with nausea and vomiting accompanied by systemic features of infection. She reports a recent episode of cellulitis of the right index finger due to injury by a thorn. She was treated with cephalexin and clindamycin for 10 days, followed by Bactrim and clindamycin as prescribed by her primary care physician. She reports beginning the Bactrim one week prior to admission. She also reports the occasional use of naproxen, unrelated to her recent cellulitis. On presentation, her blood pressure was 174/103, heart rate was 96, and temperature was 37.4 C. A mild, linear papular rash was noted across the midline of the abdomen (Figure 1). The papules were approximately 2-3 mm in size and were non-pruritic and non-blanchable. Lab results included an elevated troponin (2.380), aspartate aminotransferase (176), and alanine transaminase (132). WBC was 12.2 with neutrophils (88.8) and eosinophils (0.5%) (Table 1, 2). EKG showed no ischemic changes (Figure 2). Cardiology was consulted due to the elevated troponins (Table 3). Echocardiography demonstrated diffuse hypokinesis and an ejection fraction of 55% (Video 1). Cardiac catheterization revealed normal coronary arteries. At this time, the cause of her symptoms was suspected to be systemic infection due to the progression of her cellulitis as she met systemic inflammatory response syndrome (SIRS) criteria, had a suspected source of infection, and signs of organ dysfunction. She was started on broad-spectrum antibiotics as well as intravenous fluids.

**Table 1:** Laboratory values on admission BUN - blood urea nitrogen, AST - aspartate aminotransferase, SGOT - serum glutamic-oxaloacetic transaminase, ALT - alanine transaminase, SGPT - serum glutamate pyruvate transaminase, MCV - mean corpuscular volume, PLT - platelet count

Parameter	Patient values	Reference values
Na (mmol/L)	124	135-145
K (mmol/L)	3.8	3.5-5.2
Cl (mmol/L)	90	95-110
CO2 (mmol/L)	23	19-34
Anion Gap (mmol/L)	11.8	5-15
Glucose (mg/dL)	141	70-110
BUN (mg/dL)	14	6-22
Cr (mg/dL)	0.82	0.43-1.13
Bilirubin, total (mg/dL)	0.5	0.1-1.2
Alkaline phosphatase (units/L)	71	20-130
AST/SGOT (units/L)	75	10-40
ALT/SGPT (units/L)	57	10-60
WBC count (10^3^/mL)	12.2	4.5-11
RBC count (10^6^/mL)	4.6	3.93-5.22
Hemoglobin (g/dL)	12.7	11.2-15.7
Hematocrit (%)	38.3	34.1-44.9
MCV (FL)	82.7	79.4-94.8
PLT (10^3^/mL)	190	150-400
Segmented neutrophils (%)	88.8	40-70
Eosinophils (%)	0.5	1-6
Lymphocytes (%)	6.4	19.3-51.7
Neutrophils, absolute count (10^3^/mL)	10.87	1.56-6.13
Eosinophils, absolute count (10^3^/mL)	0.06	0.04-0.36
Lymphocytes, absolute count (10^3^/mL)	0.78	1.18-3.74
Lactic acid (mmol/L)	2.4	0.4-2.0
Troponin I (ng/mL)	2.380	0.000-0.034

**Table 2:** Notable lab trends from day one to day seven AST - aspartate aminotransferase, SGOT - serum glutamic-oxaloacetic transaminase, ALT - alanine transaminase, SGPT - serum glutamate pyruvate transaminase

Day	Day 1	Day 2	Day 3	Day 4	Day 5	Day 6	Day 7	Reference values
Eosinophils (%)	0.5	2.3	5.7	11.2	-	13.3	13.0	1-6
AST/SGOT (units/L)	75	-	68	52	37	108	176	10-40
ALT/SGPT (units/L)	57	-	57	54	48	83	132	10-60

**Table 3:** Troponin trend from day one to day four

Day	Day 1 initial measurement	Day 1 repeat 1	Day 1 repeat 2	Day 2 initial measurement	Day 2 repeat	Day 3 initial measurement	Day 3 repeat	Day 4	Reference values
Troponin I (ng/mL)	2.380	2.120	6.410	3.900	3.520	3.280	3.180	0.514	0.000-0.034

**Video 1:** Echocardiogram

**Figure 3:**
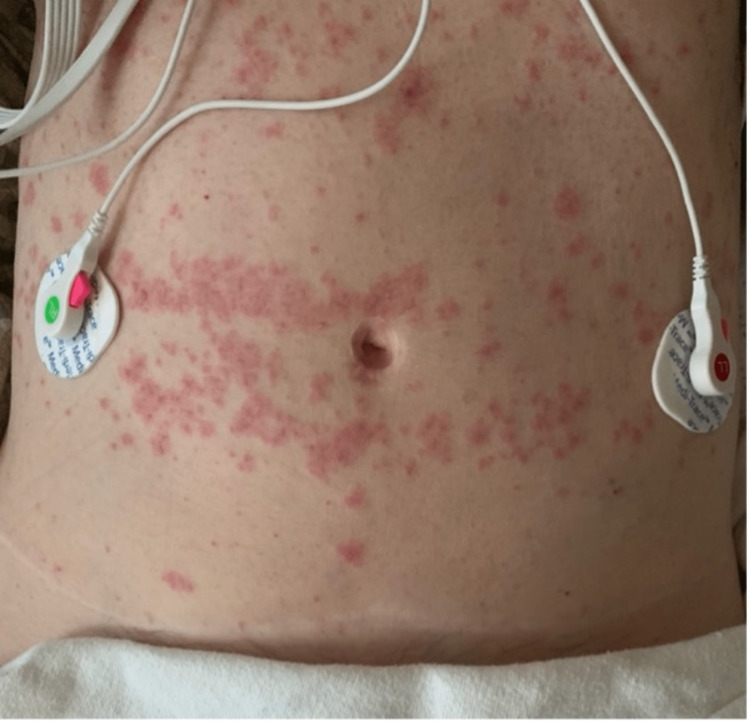
Day four of admission

**Figure 4:**
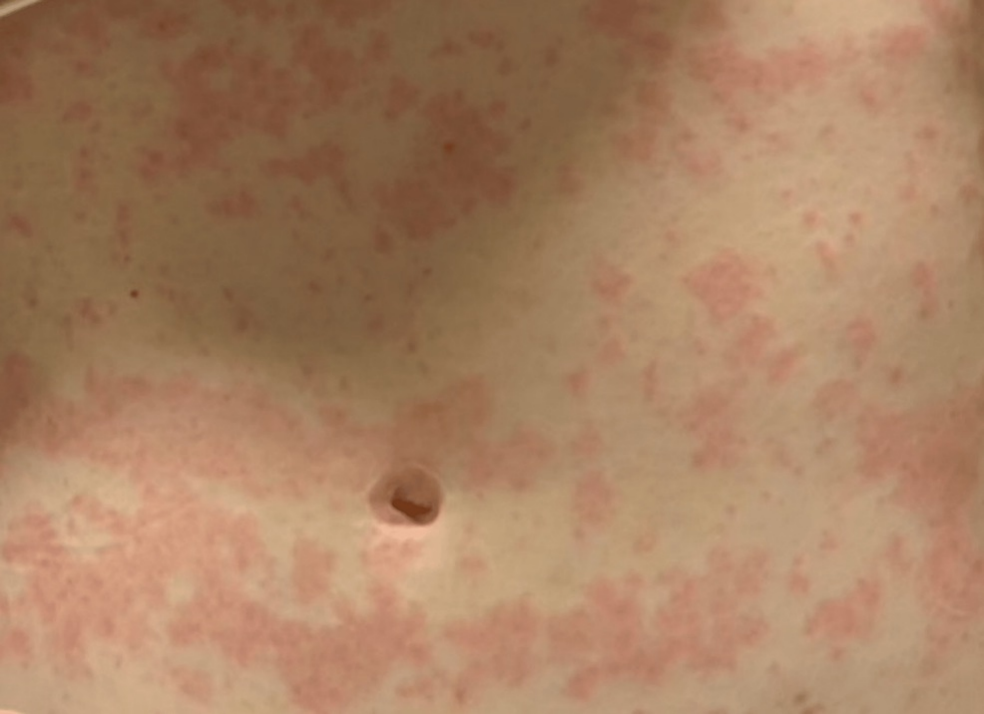
Day five of admission

**Figure 5:**
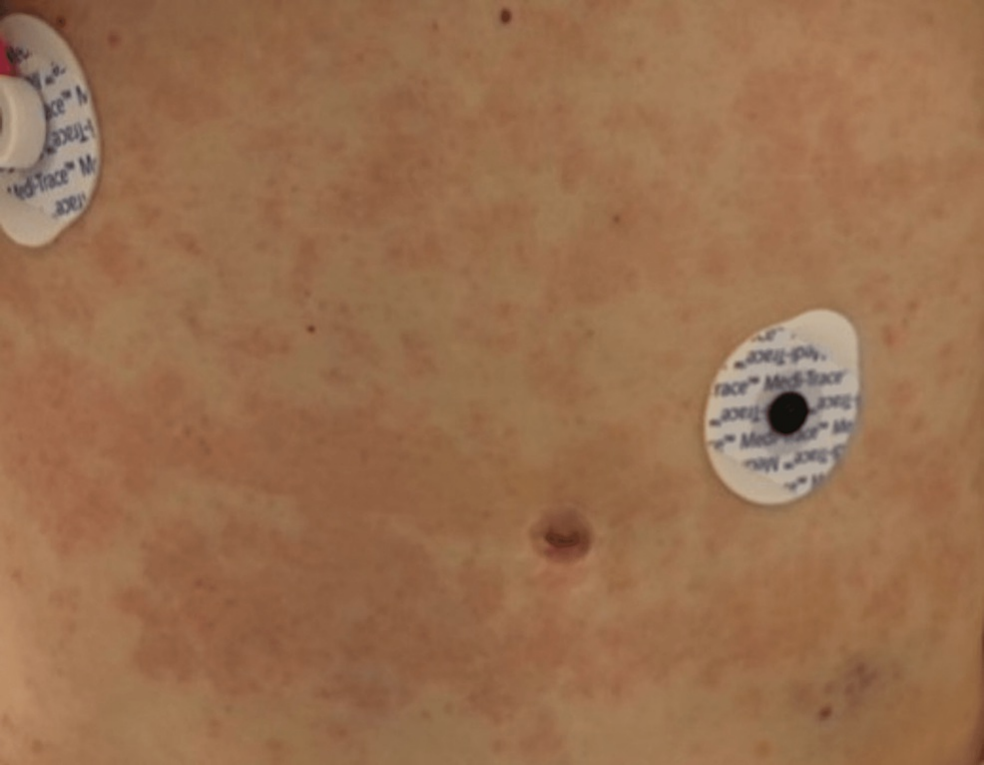
Day seven of admission

On day four, the eosinophil count increased to 11.2%. This was accompanied by the evolution of the papular rash, becoming confluent and morbilliform over her abdomen and back (Figures 3, 4). Management included discontinuation of all culprit drugs and symptomatic management with intravenous fluids and supplemental oxygen as needed. Laboratory values and vital signs were continuously monitored. Her symptoms and rash improved, and the patient was discharged on day seven (Figure 5). The patient agreed to follow up with her primary care physician within three to six days.

## Discussion

This case study reports the observation of a patient with DRESS who did not present with classic features such as eosinophilia or a typical morbilliform rash. In addition, the patient presented with severe cardiac involvement, which is not a common feature of DRESS [7]. These atypical clinical features, along with her recent cellulitis, provided lower suspicion for DRESS at presentation. DRESS should be considered when a patient reports prior use of medications such as sulfonamide-containing antibiotics, anticonvulsants, vancomycin, or NSAIDs, even when classic clinical features are absent [8]. Timely diagnosis and subsequent treatment of DRESS is crucial in preventing unfavorable outcomes such as multiple organ failure and death. DRESS has a mortality rate of up to 6-10%, while visceral organ involvement evokes a higher risk of mortality, with cardiac involvement evoking a risk of 45.2% [7,9,10]. Our patient presented with mild hepatic involvement and likely hypersensitivity myocarditis without eosinophilia or characteristic rash, contributing to a delay in diagnosis [11].

The diverse presentation of DRESS provides clinicians with a challenge in diagnosis. Additionally, there is a lack of abundant literature regarding such cases, which further fails to provide clinicians with evidence when there is suspicion of an atypical presentation of DRESS. The purpose of this case report is to add to the existing literature regarding atypical presentations of DRESS and to encourage clinicians to maintain a high index of suspicion of DRESS when a patient who has taken a possible offending drug presents with systemic symptoms and has an evolving illness including worsening rash, eosinophilia, and visceral organ involvement.

## Conclusions

Although DRESS is rare, it may progress to multiple organ failure and death, making identification of clinical features and establishing a diagnosis critical. When patients present atypically, it is challenging to establish an initial diagnosis which may lead to delayed cessation of the offending drug and subsequent resolution of symptoms. Because DRESS may present with diverse clinical features outside the classic presentation, a thorough medical history is essential. For this reason, the authors of this case report encourage clinicians to consider DRESS as part of the differential diagnosis anytime a patient has a history of use of an offending drug, even if the presentation does not appear to be DRESS initially. Maintaining clinical suspicion of DRESS is of the utmost importance in achieving the desired prognosis and decreasing life-threatening risk.
